# Exploring and Identifying Candidate Genes and Genomic Regions Related to Economically Important Traits in Hanwoo Cattle

**DOI:** 10.3390/cimb44120414

**Published:** 2022-12-04

**Authors:** Masoumeh Naserkheil, Zeinab Manzari, Chang Gwon Dang, Seung Soo Lee, Mi Na Park

**Affiliations:** 1 Animal Breeding and Genetics Division, National Institute of Animal Science, Cheonan-si 31000, Republic of Korea; 2 Animal Genetics and Breeding Unit, University of New England, Armidale, NSW 2351, Australia

**Keywords:** genome-wide association, candidate gene, SNP, economically traits, Hanwoo

## Abstract

The purpose of the current review was to explore and summarize different studies concerning the detection and characterization of candidate genes and genomic regions associated with economically important traits in Hanwoo beef cattle. Hanwoo cattle, the indigenous premium beef cattle of Korea, were introduced for their marbled fat, tenderness, characteristic flavor, and juiciness. To date, there has been a strong emphasis on the genetic improvement of meat quality and yields, such as backfat thickness (BFT), marbling score (MS), carcass weight (CW), eye muscle area (EMA), and yearling weight (YW), as major selection criteria in Hanwoo breeding programs. Hence, an understanding of the genetics controlling these traits along with precise knowledge of the biological mechanisms underlying the traits would increase the ability of the industry to improve cattle to better meet consumer demands. With the development of high-throughput genotyping, genomewide association studies (GWAS) have allowed the detection of chromosomal regions and candidate genes linked to phenotypes of interest. This is an effective and useful tool for accelerating the efficiency of animal breeding and selection. The GWAS results obtained from the literature review showed that most positional genes associated with carcass and growth traits in Hanwoo are located on chromosomes 6 and 14, among which *LCORL, NCAPG, PPARGC1A*, *ABCG2*, *FAM110B*, *FABP4*, *DGAT1*, *PLAG1*, and *TOX* are well known. In conclusion, this review study attempted to provide comprehensive information on the identified candidate genes associated with the studied traits and genes enriched in the functional terms and pathways that could serve as a valuable resource for future research in Hanwoo breeding programs.

## 1. Introduction

In recent decades, selective breeding programs have focused on improving meat production in beef cattle as the need for high-quality animal protein is growing throughout the world [1]. Carcass and growth traits are economically important traits for beef cattle due to their influence on the profitability of the meat industry. Hanwoo (*Bos taurus coreanae*) cattle, which are indigenous and unique to Korea, are popular meat-type cattle due to their high-quality meat and rapid growth. Notably, their beef is characterized by extensive marbling and quality attributes, including tenderness, good flavor, texture, and juiciness [2]. Hanwoo cattle have been intensively bred for beef over the last four decades; however, the breed was utilized extensively for transportation, farming, and religious sacrifices in earlier times. There are some studies on the domestication and origin of this breed. It is generally accepted as an independent domestication event for north-east Asian cattle, and the history of Hanwoo cattle as draft animals dates back 5000 years. There are three main types in Korea, which are distinguished by different coat colors: brown, jeju black, and brindle [3]. The brown color is the most common and is preferred for breeding schemes of this breed. The important characteristics in the selection criteria of a Hanwoo breeding program that are commonly included in efforts to improve both the quality and quantity of the meat and have major impacts on profitability for the Korean beef industry are backfat thickness (BFT), marbling score (MS), carcass weight (CW), and eye muscle area (EMA) [4]. Currently, Hanwoo proven bulls are selected through a performance test for young bulls and through a progeny test for selected young bulls, with traits such as marbling score and yearling weight (YW) implemented for selecting young bulls, and backfat thickness, carcass weight, eye muscle area, and marbling score being used for choosing proven bulls in national Hanwoo genetic evaluations [5,6]. Hence, an emphasis on the identification of causal genes and understanding the genetic architecture underlying these traits is required to improve the efficiency of animal breeding and selection. With the advent of sequencing techniques and high-throughput genotyping and progresses in developing computational methods, genomewide association studies (GWAS) have become a powerful and affordable tool to detect and localize genomic regions and candidate genes associated with quantitative traits, not only in beef cattle, but also in several other species [7]. Moreover, supplementing a GWAS with gene-set enrichment, pathway analyses, and a protein–protein interaction network (PPI) could lead to a better understanding of the molecular mechanisms that regulate complex traits [8,9]. For constructing the PPI network, the identified candidate genes from literature mining were used to indicate the physical connection between two or more protein molecules related to biochemical functions. Briefly, PPI networks provide a good basis with which to describe protein associations. Hence, studying the relationships between proteins allows us to identify the biological pathways and the physiological mechanisms of interest. To date, numerous studies have attempted to identify major quantitative trait loci (QTL) linked to carcass and growth traits using a GWAS approach in Hanwoo and other beef cattle. Interestingly, the mapped QTLs were mostly located on chromosomes 6 and 14 [4,10,11,12]. Hence, the characterization of QTL allows a biomarker-assisted assortment of economically important traits and can further accelerate genetic gain when candidate genes are associated with traits of economic importance [13]. Furthermore, the incorporation of genome-wide SNP panels into genetic evaluation models enables us to estimate the genomic breeding value (GEBV) that results in an improved accuracy of genomic prediction and an increased selection response for the traits of interest in cattle [14]. In recent years, the importance of genomic selection as a promising tool for gains in the accuracy of predictions compared with conventional genetic methods for carcass traits in Hanwoo breeding programs have been investigated in several studies [15,16,17,18,19,20,21]. The objective of the present review is to summarize studies concerning the identification of genomic regions and respective candidate genes and their corresponding pathways connected to carcass and growth traits in Hanwoo cattle. In fact, selection for these valuable traits requires knowledge of the genetic basis and biological mechanisms controlling them, which could be helpful for future genomic evaluations targeting the improvement of these traits, and thus influencing the profitability of the beef production system.

## 2. Candidate Genes Affecting Back Fat Thickness and Marbling Score

**Figure 1:**
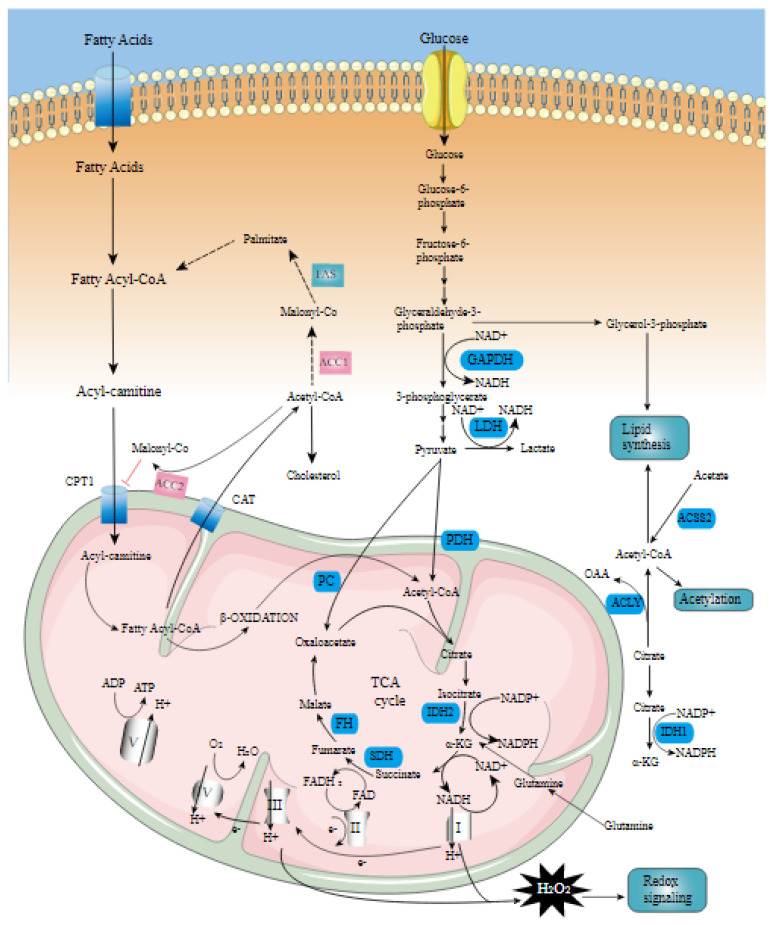
Lipid metabolism signaling pathway (https://www.creative-diagnostics.com/lipid-metabolism-signaling-pathway.htm; accessed on 12 November 2022). Lipid metabolism mainly includes triglyceride (TG) metabolism, metabolism of cholesterol and its esters, and phospholipid and glycolipid metabolism. In these metabolic processes, many proteases, receptors, transcription factors, etc. are involved, and they are regulated by some signal transduction pathways, forming a complex and fine regulatory network to maintain the lipid metabolism balance of cells and the whole body. Lipid metabolism transduction signal pathways mainly include peroxisome proliferator-activated receptor (PPAR) signal transduction pathway, liver X receptor (LXRs) signal transduction pathway, and sterol regulatory element-binding protein (SREBPs) signal transduction guide route.

Meat quality is a generic concept utilized to describe satisfaction or consumer perception of beef palatability, which is determined by multiple factors such as flavor, juiciness, tenderness, and marbling [22]. In this context, back fat thickness (subcutaneous fat) is one of the most significant attributes and has a considerable impact on meat quality by protecting the muscle from cold-shortening that occurs in the carcass-chilling process after the slaughter [23]. Another trait is marbling (intramuscular fat), which is known to be a main factor influencing the sensory meat quality. These traits are influenced by several factors, including breed, genetic, age, management, feeding regime, and growth stages [24]. Moreover, in efforts to improve meat quality, earlier studies have focused on the detection of key genes along with a precise understanding of the underlying biology of quantitative traits to better meet consumer expectations [1,25,26,27,28]. Hence, the screening of potential candidate genes to comprehend the connection between gene variations and back fat thickness and intramuscular fat is necessary. The *ANGPTL3*, *DGAT1*, *FASN*, *LPL*, *SCD*, *FABP3*, *FABP4*, *STAT6*, and *MAP2K6* genes are some candidate genes that have been demonstrated to be involved in regulating fat deposition and lipid metabolism (Table 1). One of the important parameters of meat quality is fatty acid (FA) composition, which affects the firmness of adipose tissue, and the associations of five well-known candidate genes (*FABP3*, *FABP4*, *FASN*, *SCD*, and *DGAT1*) with FA composition in beef cattle have been reported previously. The FABPs are members of a family of fatty acid-binding proteins, classified as FABP1–FABP9 [29], that play an essential role in the metabolism of long-chain fatty acids, transport, and uptake. Among them, the *FABP3* and *FABP4* genes have been shown to be related to marbling and back fat thickness in Hanwoo cattle. The *FABP3* (fatty acid-binding protein 3) gene, also known as heart-type FABP, is involved in the transport of fatty acid from the plasma membrane to intracellular sites [30] and is mainly expressed in tissues, including lactating mammary gland, skeletal muscles, heart muscle, and adipose tissues [31]. In bovine, this gene, located at 122 Mb on chromosome 2, contains 4 exons [32], and its function is to bind unsaturated long-chain and non-esterified saturated fatty acids and other lipids for storage or transportation inside a cell [33,34]. The results of gene expression analysis revealed that the *FABP3* gene was down-regulated in the high-marbled compared to the low-marbled group of Hanwoo cattle [35]. In another study, it has been found that variation in the *FABP3* gene was related to back fat thickness in Hanwoo cattle [36]. The *FABP4* (fatty acid-binding protein 4) gene, mapped on chromosome 14 and encoding adipocyte fatty acid-binding proteins, is mainly expressed in adipocytes [37]. This gene has a key function in lipid catabolism through changes in the intracellular targeting of fatty acids and lipid hydrolysis [38], and is involved in the *Peroxisome Proliferator-Activated Receptor (PPAR)* signaling pathway. It was suggested that the *PPAR* signaling pathway is related to energy metabolism, regulating adipocyte tissue development, and lipogenesis. Moreover, it has been shown that the *FABP4* gene is associated with back fat thickness [36], fatty acid composition [39,40], and marbling score [41,42] in Hanwoo cattle. Another interesting finding by Lim et al. [35] showed that the *FABP4* gene was up-regulated in muscle with high marbling levels in Hanwoo cattle. The bovine *FASN* (fatty acid synthase) encodes a complex homodimeric enzyme involved in the regulation of long-chain fatty acid biosynthesis [43], and is extensively expressed in the adipose tissue. The *FASN* gene has seven active sites and catalyzes the formation of fatty acids of palmitate from malonyl coenzyme A and acetyl-coenzyme A [43]. This gene is a candidate gene located in the position of 50 Mb on chromosome 19, and it has been related to FA composition in various beef cattle breeds [44,45]. Mutations in the *FASN* gene have been reported in several studies of Hanwoo cattle. It was shown that five exonic SNPs (g.12870 T > C, g.13126 T > C, g.15532 C > A, g.16907 T > C, and g.17924 G > A) in the gene encoding *FASN* were connected to FA composition and marbling score [46]. In addition, another study reported 16 polymorphisms, six of which were nonsynonymous mutations in the *FASN* coding regions in Hanwoo cattle [47]. Furthermore, two GWAS studies identified that this gene was significantly associated with C14:0 [48] and marbling score [41]. A positional candidate gene on bovine chromosome 14 is *DGAT1* (diacylglycerol O-acyltransferase 1), a microsomal enzyme that catalyzes the final step of triglyceride synthesis [49]. Substitution of lysine for alanine in exon 8 region 232 (K232A) of this gene has been proposed to be associated with fat content and composition [50,51], marbling score [52], intramuscular fat content [53,54,55], and subcutaneous fat thickness [56] in different cattle breeds. This gene is related to transferase activity, transferring phosphorus-containing groups, and 2-acylglycerol O-acyltransferase activity, according to gene ontology (GO) terms, and it is involved in different pathways, including metabolic pathways, fat digestion and absorption, and glycerolipid metabolism. Regarding fatty acid biosynthesis in cattle, the *SCD* (stearoyl-CoA desaturase) gene encodes an enzyme that catalyzes the conversion of saturated fatty acid (SFA) to monounsaturated fatty acid (MUFA), primarily the synthesis of oleic acid in adipose tissue [57]. The bovine *SCD* gene is mapped on chromosome 26 with six exons, and plays an essential role in regulating the expression of genes that are involved in lipogenesis as well as regulating mitochondrial fatty acid oxidation and body energy homeostasis. Candidate gene studies in Hanwoo cattle have reported that polymorphisms in the *SCD* gene have important effects on marbling score, meat texture, and grade of meat quality [41,58,59,60,61,62]. The *STAT6* (signal transducer and activator of transcription 6) gene, located on chromosome 5 and identified to be linked to back fat thickness, was previously shown to be associated with carcass weight, calculated yield grade, cutability, back fat rate, dry matter intake, days on feed, and average daily gain in different beef cattle breeds [63]. This gene acts both as a mediator of leptin signaling and as a transcription factor, playing an essential role in the regulation of body weight by signaling the size of adipose tissue mass [64,65]. In addition to the above-discussed genes, *ANGPTL3*, *LPL*, and *MAP2K6* genes were also found to be involved in regulating the lipid metabolism signaling pathway (Figure 1). *ANGPTL3* (angiopoietin-like 3) gene is a member of the ANGPTL family that plays a crucial role in lipid metabolism by enhancing high-density lipoprotein cholesterol and plasma lipids [66], and free fatty acids (FFA) transport in adipose tissue [67]. The bovine *LPL* (lipoprotein lipase) gene, located on chromosome 8 with ten exons, encodes a key enzyme in triglyceride metabolism and has the dual functions of triglyceride hydrolase and ligand/bridging factor for receptor-mediated lipoprotein uptake [68,69]. Previous studies reported that the *MAP2K6* gene was associated with marbling score and back fat thickness in Hanwoo cattle [11,70] and fatty acid profile in Nellore cattle [71]. This gene is a member of the dual-specificity protein kinase family that plays a role in regulating the mitogen-activated protein kinase pathway.

## 3. Candidate Genes Affecting Carcass Weight

One of the most important quantitative traits that can affect the meat production system is carcass weight. This trait is controlled by both genetic and environmental factors [75]. A number of genes have been identified as potential candidate genes related to carcass weight in Hanwoo cattle, among which *SDCBP*, *CYP7A1*, *TMEM68*, *TOX*, *FAM110B*, *UBXN2B*, *PPARGC1A*, and *GHR,* mostly located on chromosome 14, were noteworthy (Table 2). For instance, *SDCBP* (syndecan-binding protein), mapped on a conserved region on chromosome 14, was previously reported as a candidate gene for carcass weight in Hanwoo cattle [4,9,10]. It encodes a protein that binds to a variety of transmembrane proteins. This protein may also influence cell adhesion, cytoskeletal-membrane organization, the activation of transcription factors, and protein trafficking. The bovine *CYP7A1* gene, which is a member of cytochrome P450, family 7, subfamily A, polypeptide 1, was identified in the position of 24 Mb on chromosome 14 and has five introns and six exons. This gene encodes a rate-limiting enzyme during bile acid synthesis that is involved in the synthesis, secretion, and transport of cholesterol in the liver [76] and participates in lipid metabolism and maintaining the balance of cholesterol within the body [77]. It has been demonstrated that the *CYP7A1* gene is linked to carcass weight and could be considered as a biomarker for selection in Hanwoo cattle [4,9,10]. In addition to these genes, *TMEM68*, *TOX*, *FAM110B*, and *UBXN2B*, positioned in the region spanning from 23 to 25 Mb on chromosome 14, have been previously identified to affect several traits, such as growth, birth weight, carcass weight, average daily gain, feed intake, meat tenderness, height, and stature, in different beef cattle breeds [26,78,79,80]. *TMEM68* (transmembrane protein 68) encodes an enzyme that catalyzes important reactions such as those involving proteins, lipids, or other substrates located within or near the fat layers [81]. This gene is expressed in the adipose tissue, rumen, intestine, and abomasum in cattle, and is involved in lipid biosynthetic processes. The *TOX* gene is composed of nine exons and is implicated as a key transcription factor (TF) in regulating puberty in beef cattle [82]. Previous GWAS studies indicated that this gene was associated with carcass weight in Hanwoo cattle [4,10,11,83,84,85]. Furthermore, the QTL for carcass weight in Hanwoo cattle was detected around the *FAM110B* gene (family with sequence similarity 110, member B), which is a protein-coding gene involved in cell cycle progression and responsible for increasing cell number and cell size [4,11,12,84,85]. Another positional candidate gene, *UBXN2B* (UBX domain protein 2B), known as a protein-coding gene consisting of eight exons, is related to carcass weight in Hanwoo cattle [9,11,83,84]. This gene is involved in endoplasmic reticulum biogenesis and is annotated into ubiquitin binding and protein phosphatase regulator activity in terms of gene ontology. The most interesting candidate gene identified on bovine chromosome 6 for carcass weight is the *PPARGC1A* gene, also known as *PGC-1α* [86]. The *PPARGC1A* gene (peroxisome proliferator-activated receptor gamma coactivator-1 alpha) encodes a transcriptional coactivator, which regulates genes involved in mitochondrial biogenesis as well as lipid and glucose metabolism [87,88], and is expressed primarily in tissues of high metabolic activity, such as liver, adipose tissue, heart, and exercising oxidative skeletal muscle fibers [89]. Indeed, it was found that *PPARGC1A* is a metabolic switch and convergently regulates metabolic pathways via its pleiotropic interactions with nuclear receptors (NRs) and other non-NR transcription factors, such as thyroid hormone receptors (TRs), peroxisome proliferator activated receptors (PPARs), nuclear respiratory factors (NRFs), CCAAT element-binding proteins (C/EBPs), estrogen-related receptors (ERRs), and the sterol regulatory element-binding protein (SREBP) [86]. Several polymorphisms have been detected in *PPARGC1A* gene, and significant relationships between SNPs of this gene and carcass weight have been reported in Hanwoo cattle [10,12,41,90]. *GHR* (growth hormone receptor) is located on chromosome 20 in cattle and consists of 10 exons and nine introns [91,92], and it has been considered as a candidate gene for meat quality and growth traits [93,94]. *GHR* is a member of the class I cytokine receptor family and is bound by growth hormone (GH), leading to the activation of hormonal systems involved in growth development [95]. This protein is a 638-amino acid-long homodimeric receptor with one cytokine receptor homology domain (CRH), a cytoplasmic intracellular domain (ICD), and a single-pass transmembrane domain. It was reported that mutation of the *GHR* gene may affect the signaling pathway and its binding capacity, thereby resulting in alteration of the GH activity in the target tissues [93]. An SNP in the *GHR* gene was identified as being associated with carcass weight, lean cuts, carcass dressing percentage weights, and growth traits in Hanwoo cattle [41] and other breeds [96,97]. Further functional analysis showed that the identified genes are involved in several biological processes, such as cellular response to chemical stimulus, regulation of the lipid metabolic process, and the catabolic process.

## 4. Candidate Genes Affecting Eye Muscle Area

Another important meat quantity trait is eye muscle area (EMA), which is the area of the surface of the longissimus dorsi muscle between the 12th and 13th ribs and a relevant indicator of the meat yield, carcass muscularity, and carcass weight [23,98]. It is a significant factor in the determination of carcass yield grade, since as the eye muscle area increases, so does the amount of lean muscle in a carcass. According to the results of literature mining of Hanwoo cattle, a total of seven candidate genes, including *CAPN1*, *CAST*, *LEP*, *LEPR*, *IGF1*, *IGF2*, and *MSTN,* were most relevant to this trait (Table 3). In cattle, some polymorphisms of the *CAPN1* and *CAST* genes were found to be responsible for meat tenderness of skeletal muscle [54,99,100,101,102]. CAPN1/CAST is an endogenous calcium-dependent proteinase system that mediates the proteolysis of key myofibrillar proteins during the postmortem period [103]. The bovine *CAPN1* (calpain 1) gene, mapped to chromosome 29 with 24 exons, encodes the large subunit of calcium-activated neutral proteases as an enzyme involved in the tenderization process [99,104]. This enzyme degrades myofibrillar proteins postmortem, controls the function of weakening the bonds between bundles of muscle fibers, owing to the decomposition of the Z-disks of skeletal muscle of calcium-dependent cysteine protease, and creates the conditions for a uniform distribution of intramuscular fat between the fibers [105]. The *CAST* (calpastatin) gene is found on bovine chromosome 7 and contains 39 exons, and it regulates the proteolytic activity of calpains [28]. It was shown that the serine/threonine substitution (S20T) in the *CAST* gene is connected to carcass traits in Simmental cattle [96]. Moreover, the associations of seven SNPs in *CAPN1* and *CAST* genes with meat tenderness have previously been reported in two different muscle cuts in Hanwoo cattle [25]. *LEP* and *LEPR* genes were identified as promising candidate genes responsible for increasing weight, growth traits [106,107], energy storage [106,108], and lipid metabolism [109]. The bovine *LEP* (leptin) gene is mapped to chromosome 4 and comprises three exons and two introns, and it is mainly secreted by white adipose tissue [110] and mediated by the expression of *LEPR* (leptin receptor) in the hypothalamus [111]. The *LEPR* gene is found on bovine chromosome 3 with 21 exons and has a crucial role in the regulation of energy homeostasis and fat deposition. In a previous study, four significant SNPs in the *LEPR* gene were associated with fat deposition in Chinese beef cattle [112]. Concerning the leptin gene, several SNPs have been previously identified to be related to feed intake, fat thickness, marbling, and eye muscle area in different beef cattle breeds [113,114,115,116]. In Hanwoo cattle, Kong et al. [117] reported the association of the two SNPs (Kpn2 I and Msp I) in the *LEP* gene with ultrasound measures of carcass traits and observed that genotype CC of the Kpn2 I had a significantly higher impact on EMA than genotype TT did. In addition to this, *IGF1* and *IGF2* are also known to be associated with muscle development [118,119]. Insulin-like growth factor (IGF) has a key function in metabolism regulation, growth, and cell differentiation [120]. The bovine *IGF1* gene (insulin-like growth factor 1), also known as somatomedin, is a member of a family of proteins involved in mediating growth and development. This gene also acts as a mediator in various biological and metabolic processes, such as stimulating myogenesis, inhibition of apoptosis, the activation of cell cycle genes, increasing the absorption of glucose, increasing the synthesis of lipids, and cell proliferation [121]. Moreover, the *IGF2* (insulin-like growth factor 2) gene is mapped on bovine chromosome 29, which encodes a member of the insulin family of polypeptide growth factors and is associated with cattle muscle mass and body size. Gene ontology analysis demonstrated that *IGF1* and *IGF2* genes are involved in growth factor activity, integrin binding, and Ras–MAPK pathways. The bovine *MSTN* (myostatin) gene is a candidate gene located on chromosome 2, and it regulates both skeletal muscle mass and fiber-type composition. This gene is a member of the TGF-beta (transforming growth factor-beta) family of proteins that negatively acts to inhibit the differentiation of muscle fibers and growth [122,123]. In a previous GWAS study conducted by Lee et al. [41], the genetic variation of *MSTN* gene on carcass traits was identified in a Hanwoo cattle population. Moreover, it has been revealed that a mutation in *MSTN* resulted in double muscling in different cattle breeds [124,125,126].

## 5. Candidate Genes Affecting Yearling Weight

Yearling weight represents an important meat quantity trait as it is one of the significant indicators affecting growth performance, and thus receives substantial attention in many performance-testing programs. Yearling weight has previously been documented as a moderately heritable trait in Hanwoo cattle and was shown to be strongly genetically correlated with carcass weight [18,136]. Hence, given the GWAS results in the literature, a better understanding and exploration of the candidate genes and QTL explaining the large effect on this trait is required. Previous studies demonstrated that genomic regions on bovine chromosomes 6 and 14 containing seven candidate genes, namely, *ABCG2*, *NCAPG*, *LCORL*, *CHCHD7*, *PLAG1*, *ANXA13*, and *PENK,* were putatively linked to growth-related traits (Table 4) in both dairy and beef cattle breeds [137,138,139,140,141,142,143]. The *ABCG2* (ATP-binding cassette subfamily G member 2) gene is a member of the White subfamily, which participates in iron transportation and metabolism, and has a key function in protein homodimerization activity and ATP hydrolysis activity. This gene has been previously linked to direct birth weight, weaning weight, and yearling weight in Brangus cattle [144] and also primal cut yields [145], carcass weight, and yearling weight in Hanwoo cattle [12]. The significant association of causative variations of *NCAPG* and *LCORL* genes with yearling weight and growth traits was found in various beef cattle breeds. In bovine, the *NCAPG*–*LCORL* locus is located on chromosome 6 between 37.30 and 37.55 Mb on the ARS-UCD1.2 genome assembly. The *NCAPG* (non-SMC condensin I complex subunit G) gene encodes a protein of the mammalian condensin I complex and is involved in the stabilization and condensation of chromosomes, as well as having important roles in modulating fetal growth [146] and regulating mitotic cell division [147]. It has previously been reported that the p.I422M polymorphism in *NCAPG* was linked to growth in three populations of cattle breeds [140,146]. The importance of this mutation was evident in a study by Weikard et al. [148], where it was related to linoleylcarnitine (C18:2) levels, symmetric dimethylarginine (SDMA), and circulating levels of plasma arginine. It was suggested that arginine influences growth and more particularly the growth of skeletal muscle by activating the mTOR signaling pathway (bta04150) [149]. This pathway is known to regulate several biological processes, including lipid metabolism, protein synthesis, autophagy, and ribosome biogenesis. Furthermore, this gene has a role in increasing carcass yield and decreasing subcutaneous fat thickness [150]. Similarly, there is evidence to indicate that the *LCORL* gene has an important influence on growth and skeletal frame size [151,152,153]. The *LCORL* (ligand-dependent nuclear receptor corepressor-like) gene encodes a transcription factor that interacts with ubiquitin C [154]. In addition to this, the *LCORL* gene has been associated with arginine metabolism in growth [148,155]. Interestingly, the *NCAPG* and *LCORL* genes are known as pleiotropic genes and have been previously linked to carcass weight and body frame size for Japanese Black cattle [140,141], with yearling weight, birth, and weaning weight in crossbred beef cattle [142], and with subcutaneous fat thickness, body weight gain, and feed intake in 14 different breeds of cattle [156]. Importantly, the most significant candidate genes for yearling weight and growth traits were located on bovine chromosome 14, as reported above. It was shown that several causal variants found in the well-known *PLAG1*–*CHCHD7* position affected the regulation of stature and growth in cattle [26,139,157,158,159]. The *PLAG1* gene (pleomorphic adenoma gene 1) is a member of the PLAG family of zinc finger transcription factors, and it has a certain regulatory role in mammalian growth and body weight [158]. This gene encodes a multifunction transcription factor and regulates several growth factors controlling body size, such as bone-derived growth factor (BPGF1), insulin-like growth factor II (IGF2), and cytokine-like factor 1 (CLF1) [160]. Moreover, the *CHCHD7* (coiled-coil-helix-coiled-coil-helix domain-containing 7) gene has been demonstrated to be a possible causative gene for bovine stature. This gene consists of five exons and participates in signaling pathways, such as mitochondrial protein import and metabolism of proteins. Furthermore, some studies showed that the *CHCHD7* gene is responsible for shank, biceps, and knuckle traits in Chinese Simmental cattle [161], carcass weight in Japanese Black [159] and Hanwoo steers [4,9,12], and intramuscular fat and fat thickness in Nellore [23] and composite cattle [27]. *ANXA13* (annexin A13) is another important gene associated with yearling weight that encodes a member of the annexin family and has three exons. This gene is known to be involved in the regulation of cellular growth and in signal transduction pathways [162]. The *ANXA13* gene aids in the calcium-dependent phospholipid-binding protein family, which was enriched in GO terms related to calcium ion binding and phosphatidylserine binding. A pleiotropic candidate gene, *PENK* (proenkephalin), was detected on chromosome 14 at approximately 23 Mb and correlated with birth weight [143], yearling weight [12], and intramuscular fat and fat thickness [27] in different breeds of cattle. *PENK* participates in numerous physiologic functions and encodes a preproprotein, which is proteolytically processed to make multiple protein products. This gene is responsible for the regulation of GnRH [163,164], as the GnRH signaling pathway is involved in the characterization of the different stages of growth until puberty in cattle [165].

## 6. Protein–Protein Interaction (PPI) Network and Identification of Hub Genes

**Figure 2:**
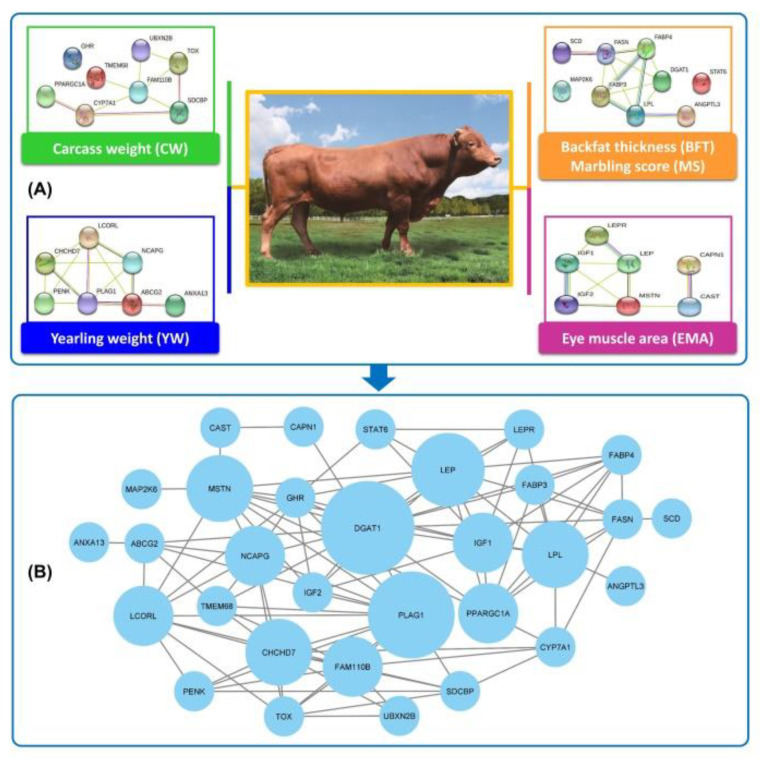
Protein–protein interaction (PPI) network analysis using STRING database. (**A**) PPI network analysis for genes connected to each trait in Hanwoo cattle. (**B**) The most significant hub genes in the PPI network are considered according to FDR < 0.05 and higher-degree connectivity value.

Protein–protein interaction (PPI) networks have been widely applied in various fields and provide a comprehensive picture of all physical interactions detected between pairs of proteins [166]. The PPI network for the genes of interest was reconstructed by STRING database v11.0 [167] to search interactions among genes specifically in *Bos taurus* species (Figure 2). This interactive network consists of 31 nodes (genes) and 103 edges (interactions) that indicate a physical connection between two or more protein molecules connected to biochemical functions. Based on higher-degree connectivity value, six genes, *DGAT1*, *PLAG1*, *LEP*, *LPL*, *MSTN*, and *CHCHD7*, were identified as hub genes in the PPI network. In other words, a higher number of interactions between hub genes and nodes within a network may represent a greater function of that gene in the activation or repression of other genes, and hence, its impact on the traits of interest. In fact, predicting protein–protein interactions allows the identification of highly connected genes and associates them with the studied traits, helping to shed light on the common pathways as well as key mediators and regulators underlying these traits [168]. For instance, based on the results of the PPI network, hub gene *DGAT1* as central node appeared to have the highest number of connections and was involved in a variety of biological processes such as the lipid biosynthetic process, lipid homeostasis, lipid metabolic process, and regulation of biological quality.

## 7. Conclusions

Developments in sequencing technologies allowed for the identification of candidate genes and genomic regions related to economically important traits in beef cattle. This review is an attempt to summarize and discuss information on the identified respective genes and their corresponding pathways that have key roles in carcass and growth traits in Hanwoo cattle. In total, thirty-one significant candidate genes associated with the traits of interest were reviewed in this study, among which *LCORL*, *NCAPG*, *PPARGC1A*, *ABCG2*, *FAM110B*, *FABP4*, *DGAT1*, *PLAG1*, and *TOX* were noteworthy in this breed and other beef cattle populations. Hence, this information can lead to a better understanding of the genetic background and biological mechanisms regulating carcass traits. Thus, it could be useful in developing selection strategies for breeding schemes aimed at improving these traits in Hanwoo cattle.

## Figures and Tables

**Table 1:** Description and chromosomal location of the most important genes related to back fat thickness (marbling score) in Hanwoo cattle.

Gene Symbol	Gene Name	Chr	Position (bp)	Reference
*FABP3*	fatty acid-binding protein 3	2	122287099–122294639	[35,41]
*FABP4*	fatty acid-binding protein 4	14	44676559–44680947	[39,40,41,42,58,60,62,72,73]
*FASN*	fatty acid synthase	19	50776167–50794939	[41,46,47,48,58]
*DGAT1*	diacylglycerol O-acyltransferase 1	14	603981–614153	[41,52]
*SCD*	stearoyl-CoA desaturase	26	21263976–21279185	[41,58,59,60,61,62]
*STAT6*	signal transducer and activator of transcription 6	5	56325240–56340447	[41]
*ANGPTL3*	Angiopoietin-like 3	3	82994334–83003679	[41]
*LPL*	lipoprotein lipase	8	66989665–67016694	[41,74]
*MAP2K6*	mitogen-activated protein kinase kinase 6	19	61130409–61236793	[11,41,70]

**Table 2:** Description and chromosomal location of the most important genes related to carcass weight in Hanwoo cattle.

Gene Symbol	Gene Name	Chr	Position (bp)	Reference
*SDCBP*	syndecan-binding protein	14	24710921–24764191	[4,10]
*CYP7A1*	cytochrome P450, family 7, subfamily A, polypeptide 1	14	24664833–24675169	[4,9,10]
*TMEM68*	transmembrane protein 68	14	23034273–23070127	[12,85]
*TOX*	thymocyte selection-associated high-mobility group box	14	24946881–25258596	[4,10,11,83,84,85]
*FAM110B*	family with sequence similarity 110 member B	14	24291600–24433683	[4,11,12,84,85]
*UBXN2B*	UBX domain protein 2B	14	24587679–24624435	[9,11,83,84]
*PPARGC1A*	PPARG coactivator 1 alpha	6	43380463–43903480	[10,12,41,90]
*GHR*	growth hormone receptor	20	31869704–32043372	[41]

**Table 3:** Description and chromosomal location of the most important genes related to eye muscle area in Hanwoo cattle.

Gene Symbol	Gene Name	Chr	Position (bp)	Reference
*CAPN1*	calpain 1	29	43399871–43427419	[25,41,127,128,129,130]
*CAST*	calpastatin	7	96034228–96183530	[25,41,128,131,132,133]
*LEP*	leptin	4	92436837–92453660	[41,117]
*LEPR*	leptin receptor	3	79741204–79838014	[41]
*IGF1*	insulin-like growth factor 1	5	66185922–66264134	[41,133,134]
*IGF2*	insulin-like growth factor 2	29	49395153–49422469	[41]
*MSTN*	myostatin	2	6278864–6285491	[41,135]

**Table 4:** Description and chromosomal location of the most important genes related to yearling weight in Hanwoo cattle.

Gene Symbol	Gene Name	Chr	Position (bp)	Reference
*ABCG2*	ATP-binding cassette subfamily G member 2	6	36475491–36603215	[12,145]
*NCAPG*	non-SMC condensin I complex subunit G	6	37301610–37378129	[10,41]
*LCORL*	Ligand-dependent nuclear receptor corepressor-like	6	37377806–37557145	[10,41]
*CHCHD7*	coiled-coil-helix-coiled-coil-helix domain-containing 7	14	23376149–23383137	[9,12]
*PLAG1*	PLAG1 zinc finger	14	23323709–23375661	[10,12,59]
*ANXA13*	annexin A13	14	16143324–16159206	[9,12]
*PENK*	proenkephalin	14	23542355–23546761	[4,12]

## Data Availability

Not applicable.
